# Evidence for Telemedicine’s Ongoing Transformation of Health Care Delivery Since the Onset of COVID-19: Retrospective Observational Study

**DOI:** 10.2196/38661

**Published:** 2022-10-14

**Authors:** Soumik Mandal, Batia M Wiesenfeld, Devin Mann, Katharine Lawrence, Rumi Chunara, Paul Testa, Oded Nov

**Affiliations:** 1 Department of Population Health New York University Grossman School of Medicine New York University New York, NY United States; 2 Department of Technology Management & Innovation New York University Tandon School of Engineering New York University New York, NY United States; 3 New York University Leonard N Stern School of Business New York University New York, NY United States; 4 Medical Center Information Technology New York University Langone Health New York University New York, NY United States; 5 Computer Science & Engineering New York University Tandon School of Engineering New York University New York, NY United States; 6 Biostatistics, New York University School of Global Public Health New York University New York, NY United States

**Keywords:** digital health, telemedicine, urgent care, COVID-19, health care delivery

## Abstract

**Background:**

The surge of telemedicine use during the early stages of the COVID-19 pandemic has been well documented. However, scarce evidence considers the use of telemedicine in the subsequent period.

**Objective:**

This study aims to evaluate use patterns of video-based telemedicine visits for ambulatory care and urgent care provision over the course of recurring pandemic waves in 1 large health system in New York City (NYC) and what this means for health care delivery.

**Methods:**

Retrospective electronic health record (EHR) data of patients from January 1, 2020, to February 28, 2022, were used to longitudinally track and analyze telemedicine and in-person visit volumes across ambulatory care specialties and urgent care, as well as compare them to a prepandemic baseline (June-November 2019). Diagnosis codes to differentiate suspected COVID-19 visits from non–COVID-19 visits, as well as evaluating COVID-19–based telemedicine use over time, were compared to the total number of COVID-19–positive cases in the same geographic region (city level). The time series data were segmented based on change-point analysis, and variances in visit trends were compared between the segments.

**Results:**

The emergence of COVID-19 prompted an early increase in the number of telemedicine visits across the urgent care and ambulatory care settings. This use continued throughout the pandemic at a much higher level than the prepandemic baseline for both COVID-19 and non–COVID-19 suspected visits, despite the fluctuation in COVID-19 cases throughout the pandemic and the resumption of in-person clinical services. The use of telemedicine-based urgent care services for COVID-19 suspected visits showed more variance in response to each pandemic wave, but telemedicine visits for ambulatory care have remained relatively steady after the initial crisis period. During the Omicron wave, the use of all visit types, including in-person activities, decreased. Patients between 25 and 34 years of age were the largest users of telemedicine-based urgent care. Patient satisfaction with telemedicine-based urgent care remained high despite the rapid scaling of services to meet increased demand.

**Conclusions:**

The trend of the increased use of telemedicine as a means of health care delivery relative to the pre–COVID-19 baseline has been maintained throughout the later pandemic periods despite fluctuating COVID-19 cases and the resumption of in-person care delivery. Overall satisfaction with telemedicine-based care is also high. The trends in telemedicine use suggest that telemedicine-based health care delivery has become a mainstream and sustained supplement to in-person-based ambulatory care, particularly for younger patients, for both urgent and nonurgent care needs. These findings have implications for the health care delivery system, including practice leaders, insurers, and policymakers. Further investigation is needed to evaluate telemedicine adoption by key demographics, identify ongoing barriers to adoption, and explore the impacts of sustained use of telemedicine on health care outcomes and experience.

## Introduction

Before the COVID-19 pandemic, the use of telemedicine as a care delivery modality was limited; only 8% of Americans reported using telemedicine for medical care in 2019 [1]. Barriers to scaled adoption and use included limited reimbursement, patients’ and providers’ lack of comfort with telemedicine technologies, and a strong cultural norm of in-person care [2]. The dynamic interactions between these individual factors [3] often lead to nonadoption and abandonment of telemedicine technologies by their intended users [4-6]. However, beginning in March 2020, the telemedicine landscape in the United States changed rapidly, as the World Health Organization (WHO) declared COVID-19 a global pandemic, and a nationwide health care emergency was declared in the United States [7]. Prior to the large-scale availability of vaccines and effective therapies, social distancing and quarantine were the only widely accepted approaches to minimizing viral spread, creating a compelling (and often compulsory) pressure to find alternatives to in-person care [8]. To help maintain existing health care operations while meeting the new demands imposed by rising COVID-19 cases, health care systems quickly turned to telemedicine solutions for care provision, with many experiencing early exponential growth in telemedicine adoption [8]. To ensure the pace of scaling telemedicine capacity matched the growing demand, implementation with rapid iterative improvements was preferred over perfect execution [9]. Where possible, existing technology and vendors were used instead of investing time into procuring brand-new technology. As a result, telemedicine infrastructure often spanned multiple technologies and platforms, supported different modalities (voice based over the telephone, or video based) rather than any standardized implementation, and evolved rapidly in a short period, all of which could negatively affect patients’ satisfaction [10] and patients’ continued use of telemedicine.

Although research has documented the enthusiastic adoption of telemedicine technology in the early stage of the pandemic, little subsequent research has explored whether this migration has been sustained in the postacute pandemic periods. Additionally, prior reports relating the growing prevalence of telemedicine to a steady decline in in-person clinic visit volumes have suggested that telemedicine is at least partially replacing clinic visits [8]. Since the early phase of the pandemic in the spring of 2020, improved public health measures, breakthrough developments in vaccine research, and widespread vaccine and treatment protocols have made the resumption of in-person activities possible, including the provision of in-person medical care; currently, it is unclear whether the rise in telemedicine will be sustained as the US health care system transitions to more “regular” operations. There is a growing general literature on the long-term sustainability of technology-supported change in health care services [11], but studies on sustainability of telehealth services remain sparse [12]. This study, drawn from a large academic health care system in New York City (NYC), aims to explore patterns in patients’ use of telemedicine during the recurring waves of the pandemic.

The research question being answered in this study is, What were the trends in the use of video-based telemedicine visits for ambulatory care and urgent care provision over the course of recurring pandemic waves?

## Methods

### Study Setting

In this study, we used data from the New York University Langone Health (NYULH) system, a large urban and suburban academic health care system in NYC whose operations were significantly impacted by the COVID-19 pandemic and that responded by developing a robust telemedicine infrastructure to provide patient care during the period of clinic closures and disruptions.

The NYULH network consists of over 8000 health care providers across 4 hospitals and more than 350 ambulatory care locations in urban and suburban settings, all connected to a single electronic health record (EHR) system (Epic, Verona, WI). To enable its telemedicine services (known in the health system as “virtual health”), the NYULH uses a single instance of the Epic health record with more than 8.17 million active patients leveraging an integrated video visit platform. Prior to the COVID-19 pandemic, the NYULH implemented telemedicine capabilities in approximately 25 locations via its “virtual urgent care” (VUC) service, a video visit experience tightly integrated into its enterprise EHR and patient portal, offering same-day virtual appointments with emergency medical physicians for acute nonemergent health concerns (eg, new cough, fever). Virtual nonurgent care or ambulatory care, such as virtual primary care, was subsequently developed, offering a more comprehensive set of services, including chronic disease management, interdisciplinary care with specialists and ancillary care (eg, dieticians, therapists), and preventive care, with care handled by internal medicine or specialty clinicians.

Patients access the virtual services through the NYULH app built upon the Epic MyChart suite of patient tools and using standard Application Programming Interfaces (APIs) made available by Epic. During a telemedicine encounter, patients can begin their video visits directly through their patient portal app; the provider has to simply click a link in their EHR system to launch the visit. The provider’s click action opens a browser for the video that can be seen in tandem with the EHR in the same manner as an in-person visit. In addition, the NYULH has deployed native open scheduling technologies as well as custom features enabling simplified telemedicine access and matriculation. The NYULH uses Q-Reviews (New York), a real-time hospital review digital engagement platform to collect feedback from patients on their VUC visits.

### Study Design

In this study, we used patients’ visit information from the EHR data to characterize visit types from January 1, 2020, to February 28, 2022, representing the period of recurring waves of pandemic intensity. We used heterogeneous sources of data, including encounters, visits, diagnoses, patient satisfaction, and patients’ age, to identify the age groups that accessed care through telemedicine or in-person visits during this period. To categorize whether a telemedicine visit happened in ambulatory care or urgent care, the visit type, location, and specialty information were used.

To evaluate whether telemedicine use was skewed toward COVID-19 suspected visits, we evaluated *International Classification of Diseases 10th Revision* (ICD-10) diagnosis codes containing relevant primary respiratory and primary nonrespiratory symptoms via partial matching with 34 keywords (Table 1) [8,13,14]. This included COVID-19–related diagnosis codes, which were frequently used in the health system used prior to updated COVID-19 coding recommendations in 2020 and 2021 [15]. COVID-19 suspected visits were compared to total COVID-19 cases per day in NYC during similar periods to evaluate whether the prevalence of COVID-19–related illness among NYULH patients compared to the larger NYC population [16]. Descriptive statistics were computed to estimate rates of telemedicine visits in urgent care and nonurgent care settings. Telemedicine use for COVID-19 suspected and non–COVID-19 suspected visits were evaluated independently to assess for relationships between visit type and telemedicine use preference. A change-point detection analysis with binary segmentation [17] was used to identify changes in the visit trends over time and locate mean shifts in combined telemedicine and in-person visits. The change-point indexes were used to segment the 26 months’ visit data; each segment represented a change in the distribution of the time-ordered visit counts with respect to preceding and subsequent segments [18]. Finally, statistical properties (mean, variance) of visit counts were computed and compared between the segments for each visit type independently. The Levene test was used to evaluate the equality of variances in visit counts among time segments.

Prior studies that analyzed the demographics of patients during the initial surge in the use of telemedicine reported that telemedicine use was mostly confined to young patients [8,10]. To assess potential changes in telemedicine patient demographics throughout the study period and whether the expansion of telemedicine facilities has galvanized telemedicine adoption across a range of age groups, we also evaluated the age group of patients participating in telemedicine visits in our data. For each telemedicine visit record, we determined the patient’s age at the time of the visit and combined the records for patients who were from similar age groups. We compared the telemedicine use with the baseline population estimate from the 2020 US Census Bureau data for NYC [19] for each age group.

In addition to data collected from EHRs, patient satisfaction and engagement were captured and evaluated via a brief text message survey disseminated via Q-Reviews at the close of VUC telemedicine encounters. The survey assessed various domains, including satisfaction with the visit, likelihood to use telemedicine again, and how well the visit addressed/managed the patient’s medical needs, on 5-point scales (5=most satisfied); see Table 2. Satisfaction was assessed based on the responses to these 3 questions (α=.87), and trends in patients’ satisfaction were analyzed. The survey also asked respondents to estimate time costs/savings relative to in-person visits and how likely they would be to recommend VUC to a friend or colleague. Finally, average visits per patient were measured based on the count of unique patient identifiers in the data. Patients’ average telemedicine-based visits and in-person visits were compared with the prepandemic baseline.

To assess whether virtual health care delivery supplements or replaces in-person care, we calculated the average number of in-person and virtual visits per patient in 3 periods: a prepandemic baseline of June-November 2019, June-November 2020, and a postacute pandemic comparison of June-November 2021.

**Table 1:** Keywords used to identify COVID-19 suspected cases from ICD-10^a^ diagnostic codes.

Symptom type	Keywords
Primary respiratory	(1) COVID, (2) respiratory distress, (3) flu, (4) sore throat, (5) congestion, (6) URI, (7) pneumonia, (8) shortness of breath, (9) cough, (10) dyspnea, (11) pharyngitis, (12) bronchitis, (13) sinusitis, (14) ARDS, (15) lung infiltrates, (16) hypoxia, (17) tachypnea, (18) opacities, (19) wheezing
Primary nonrespiratory	(20) chest pain, (21) muscle pain, (22) joint pain, (23) stress, (24) headache, (25) fever, (26) bleeding, (27) swelling, (28) rash, (29) skin lesion, (30) insomnia, (31) malaise, (32) constipation, (33) anxiety, (34) depression

^a^ICD-10: *International Classification of Diseases 10th Revision*.

**Table 2:** Survey of patients’ satisfaction with VUC^a^.

Survey question	Scale
How satisfied were you with your VUC visit?	1-5
How well did the VUC visit address/manage your medical needs?	1-5
How likely are you to use VUC again?	1-5
How much time did you save by using VUC, including travel time?	<1 hour 1-2 hours 2-3 hours 3-4 hours >4 hours N/A^b^
How likely are you to recommend VUC to a friend or colleague?	1-10

^a^VUC: virtual urgent care.

^b^N/A: not applicable.

### Data Exclusion

Other than the EHR-integrated platform, the NYULH also used Webex by Cisco and telephone calls for a brief period for providing telemedicine services (<1% of all telemedicine visits), which are not included in this report.

### Ethical Considerations

We submitted the study proposal to the NYULH Institutional Review Board (IRB), for which exemption was awarded (#s21-01207). Further clarification with regard to the policies and terms of reference can be obtained from the IRB.

## Results

### Overall Trends in Telemedicine Use

**Figure 1:**
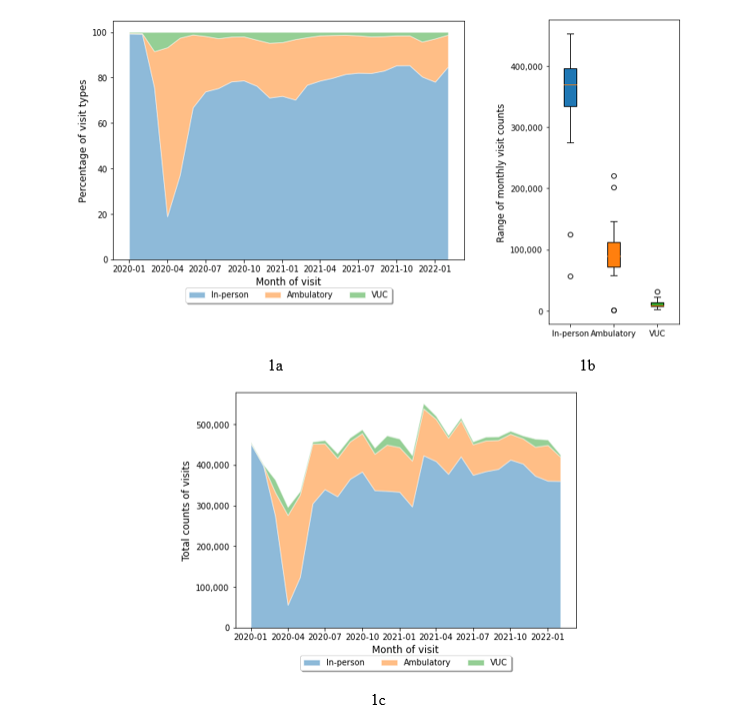
Trends in visits in telemedicine-based urgent care (VUC), nonurgent care (ambulatory), and in-person care. (a) Percentages of visit types, (b) total counts of visits per visit type, and (c) total counts for visit types per month. VUC: virtual urgent care.

During the 26-month pandemic period, a total of 2,748,635 telemedicine visits were recorded, measuring nearly one-third (30.45%) the volume of in-person visits (N=9,025,553) in the same time frame. The use of ambulatory nonurgent care (eg, virtual primary care) was much higher than VUC (see Figure 1b). Nearly 89.26% of all video visits (n=2,409,003) were for ambulatory nonurgent care (eg, virtual primary care), with the remaining 10.74% (n=289,836) visits for VUC. Overall, the visit trends showed that volumes of telemedicine visits peaked in the acute pandemic phase and continued at a higher rate than before the pandemic; telemedicine volume between January and February 2020 was <100 visits per day and subsequently peaked during the month of April 2020 (n=240,356, 80.98%) with simultaneous declines in in-person visits (Figures 1a and 1c). This shift from in-person visits to telemedicine was particularly evident during the acute pandemic period (March and April 2020) and during periods distinguished by the spread of newer strains of the virus, Delta (October 2020-January 2021) and Omicron (November 2021-January 2022). The Pearson coefficient (r) showed that volumes of in-person and VUC visits per month were negatively correlated (r=–0.421, *P*=.03). Additionally, the distribution of telemedicine visits demonstrated higher use of telemedicine by patients for nonurgent ambulatory care needs than for urgent care (see Figure 1b). Overall, the visit trends showed that volumes of telemedicine visits peaked in the acute pandemic phase, declined as in-person visits resumed, but then remained at a rate much higher than before the pandemic and with less fluctuation from July 2020 to February 2022 (monthly telemedicine visits ranged from 64,570 to 136,181 across the period). Further details of the VUC, ambulatory care telemedicine, and in-person visit data during the period are provided in Multimedia Appendix 1.

The change-point analysis detected 4 change points or mean shifts in the combined telemedicine and in-person visit trends. Based on the change-point indexes, the 26-month time series was divided into the following 5 segments: first segment (until April 2020), second segment (May-September 2020), third segment (October 2020-February 2021), fourth segment (March-September 2021), and fifth segment (October 2021-February 2022). Descriptive statistics were computed for each combination of time segment and visit type independently, and the results are provided in Table 3. Overall, the result showed the highest variations (IQR) in data on the visits per month observed in the first segment for all 3 visit types. The average number of monthly ambulatory care visits peaked in the second segment (mean 129,406, SD 46,281), which coincided with the lowest in-person visits (mean 291,829, SD 96,115). The third segment witnessed the most use (mean 16,269, SD 5351) of VUC services but with large variations (IQR 7416). The fifth and final segment was characterized by declines in both telemedicine and in-person visit types. The Levene test result found variance in visits among time segments to be significant for in-person visits (*F*_4,21_=3.56, *P*=.02) and VUC (*F*_4,21_=6.30, *P*=.001) but not for ambulatory care (*F*_4,21_=2.57, *P*=.07).

**Table 3:** Telemedicine use per month by segment.

Visit type per month	First segment (until April 2020)			Second segment (May-September 2020)			Third segment (October 2020-February 2021)				Fourth segment (March-September 2021)				Fifth segment (October 2021-February 2022)		
	Mean (SD)	Median	IQR	Mean (SD)	Median	IQR	Mean (SD)	Median	IQR	Mean (SD)		Median	IQR	Mean (SD)		Median	IQR
In person	295,607 (175,893)	336,531	191,153	291,829 (96,115)	322,933	35,107	337,981 (30,520)	336,339	3698	401,529 (23,189)		409,320	43,008	383,774 (20,086)		384,503	29,301
Ambulatory care	70,140 (103,669)	29,599	97,418	129,406 (46,281)	112,360	52,650	103,956 (11,241)	109,711	18,493	94,292 (15,399)		88,877	14,626	70,023 (9786)		71,155	10,749
VUC^a^	13,729 (14,129)	11,094	20,543	8611 (2409)	8476	1479	16,269 (5351)	15,068	7416	8028 (2693)		6802	1322	10,053 (4906)		8587	3906

^a^VUC: virtual urgent care.

### Trends in Telemedicine Service Use for COVID-19 Suspected Cases

Of all visits recorded (in person and telemedicine) in this period (N=11,774,188), 1,264,487 (~10.74%) reported at least 1 COVID-19–related symptom in the diagnosis code, representing a COVID-19 suspected case. Among these cases, 766,548 (60.62%) were recorded in in-person facilities versus 497,939 (39.38%) telemedicine visits. For COVID-19 suspected cases, the percentage of telemedicine to total visits (39.38%) was higher than the overall percentage of telemedicine to all visits (n=2,748,635, 30.45%) in the data.

**Figure 2:**
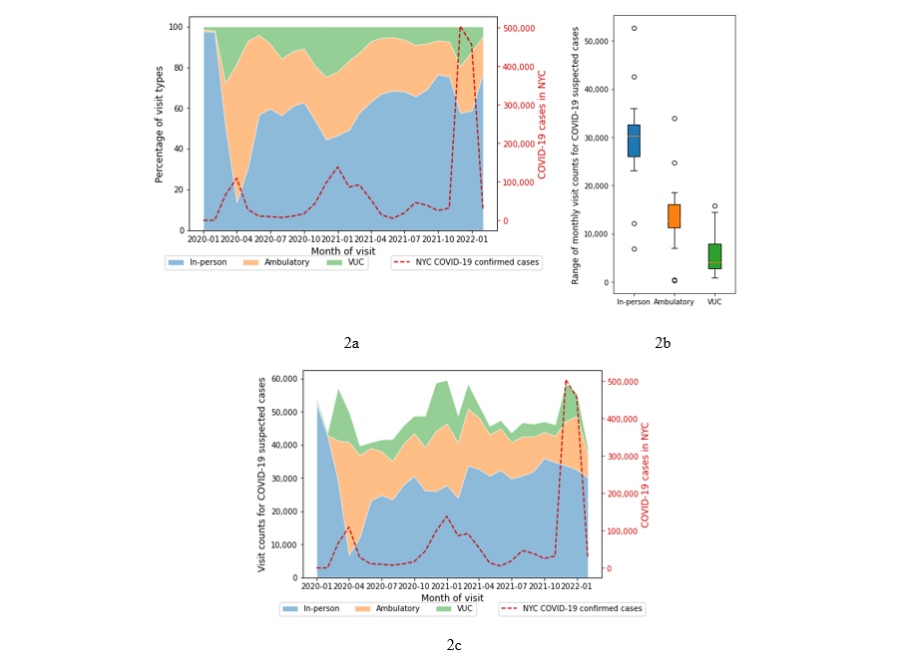
Trends in visit types for suspected COVID-19 cases and confirmed COVID-19 cases in NYC. (a) Percentages of visit types, (b) total counts of visits by visit type, and (c) total counts for visit types per month. NYC: New York City; VUC: virtual urgent care.

The distribution of video visit types for urgent and ambulatory care showed greater use of urgent care services for COVID-19 symptoms (see Figures 1b and 2b) than what was witnessed overall: the virtual visit volumes for COVID-19 suspected cases were far more evenly distributed between VUC and nonurgent facilities than what was witnessed for all recorded visits. From all COVID-19 suspected telemedicine visits, 150,735 (30.27%) were reported in urgent care facilities and the rest 347,204 (69.73%) visits in ambulatory care.

We further compared the distributions of COVID-19 suspected visit types with confirmed COVID-19 cases in NYC [20] in the same period (see Figures 2a and 2c) to evaluate the relationship between telemedicine use and surges of COVID-19 cases during recurring waves of the pandemic. The distributions demonstrated that increases in COVID-19 cases coincided with increased telemedicine visits, especially to urgent care facilities, and at the same time decreased in-person visits. This was evident in the first (March and April 2020), second (November 2020-February 2021), and third (November 2021-January 2022) waves of the pandemic, when COVID-19 cases surged in NYC. Overall, the Pearson coefficient (r) showed that counts of confirmed COVID-19 cases in NYC were negatively correlated with in-person visit volumes (r=–0.230) and almost entirely unrelated to nonurgent care visit counts (r=0.086). In contrast, urgent care visit volumes and confirmed COVID-19 numbers in NYC, which were strongly correlated (Pearson r=0.727) until November 2021, were less correlated when the Omicron outbreak was considered (r=0.393). Overall, the fraction of telemedicine visits changed more dynamically for urgent care (mean 0.15, SD 0.28 after normalization) than for ambulatory care (mean 0.85, SD 0.20) among COVID-19 suspected cases.

Table 4 shows distributions of both in-person and telemedicine service use for COVID-19 suspected cases among the 5 sequential time segments based on the change-point analysis reported before. Similar to overall visit trends, the highest variation (IQR) in visits was observed in the first segment (until April 2020) for all 3 visit types. Among the remaining 4 time segments, the highest variation in telemedicine visits was observed in the third time segment (October 2020-February 2021) for both ambulatory care (IQR 5016) and VUC (IQR 4988). Using the Levene test, the variance in COVID-19 suspected visits was found to be significant among the time segments for in-person visits (*F*_4,21_=6.55, *P*=.001) and ambulatory care (*F*_4,21_=2.85, *P*=.05) but not for VUC (*F*_4,21_=2.86, *P*=.06).

**Table 4:** Telemedicine use trends for COVID-19 suspected cases by period.

Visit type per month	First segment (until April 2020)			Second segment (May-September 2020)			Third segment (October 2020-February 2021)			Fourth segment (March-September 2021)			Fifth segment (October 2021-February 2022)		
	Mean (SD)	Median	IQR	Mean (SD)	Median	IQR	Mean (SD)	Median	IQR	Mean (SD)	Median	IQR	Mean (SD)	Median	IQR
In person	32,846 (19,777)	35,936	21,397	22,310 (5999)	23,497	1683	26,912 (2449)	26,186	1723	31,850 (1606)	32,442	2068	32,828 (2135)	32,518	2922
Ambulatory care	11,683 (15,828)	6241	17,042	15,572 (5399)	13,234	3602	15,874 (2724)	16,627	5016	13,735 (2531)	12,475	3001	10,651 (3365)	10,422	4691
VUC^a^	6,628 (7252)	4953	9,910	4014 (2015)	3485	2776	10,088 (3767)	9454	4988	3795 (2086)	2814	1172	4960 (3217)	3875	2161

^a^VUC: virtual urgent care.

### Trends in Telemedicine Service Use for Non–COVID-19 Suspected Cases

**Figure 3:**
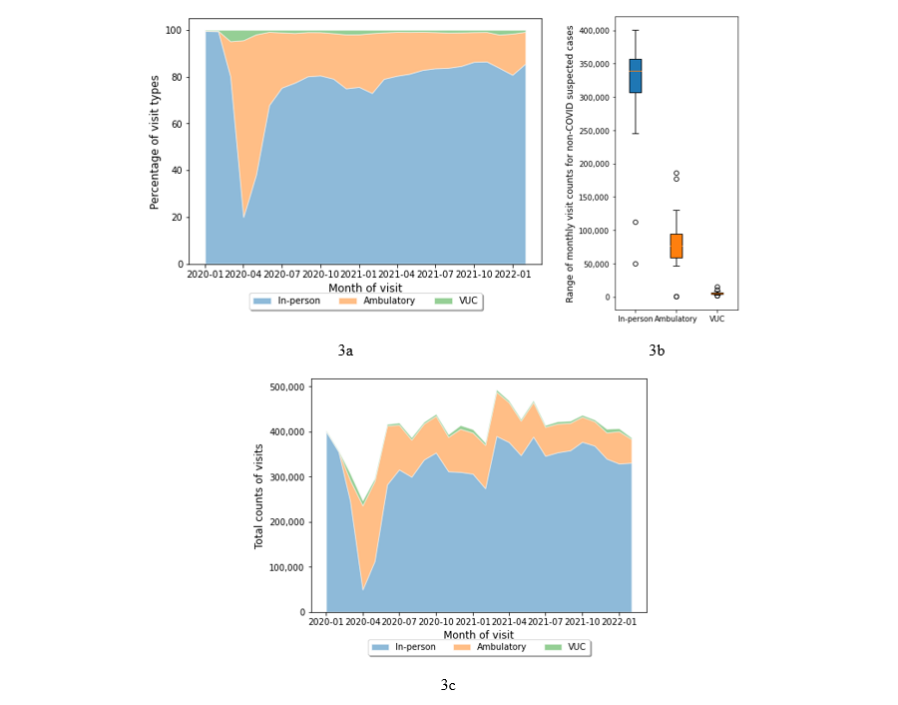
Trends in visit types for non-COVID-19 suspected cases. (a) Percentages of visit types, (b) total counts of visit types, and (c) total counts for visit types per month. VUC: virtual urgent care.

To examine the use of telemedicine beyond COVID-19 needs, we analyzed visit types for non–COVID-19 suspected cases (n=10,459,905 visits) separately (see Figure 3). Since non–COVID-19 suspected visits accounted for almost 90% of all visits recorded, their distributions across visit types (see Figures 3a and 3c) were near identical to those of all visits. For non–COVID-19 suspected cases, the proportion of telemedicine use was more skewed toward ambulatory care compared to COVID-19 suspected cases (see Figures 2b and 3b). Among all non–COVID-19 suspected telemedicine visits, nearly 2,061,799 (93.68%) cases were from nonurgent care. In the same period, only 139,101 (6.32%) non–COVID-19 suspected cases were recorded for urgent care. In addition, 8,259,005 non–COVID-19 suspected visits were in person, representing nearly 78.96% of all non–COVID-19 suspected visits from the same period. Overall, these distributions suggest that although COVID-19 prompted rapid scaling and use of telemedicine, its use grew and then remained steady at a higher level than the pre–COVID-19 baseline (Figure 3c) for non–COVID-19 suspected cases as well.

Table 5 shows variations in telemedicine use for non–COVID-19 suspected cases among the 5 segments. The distributions of monthly visits showed that among telemedicine services, the use of ambulatory care services reached its peak in the second segment (mean 113,834, SD 41,001), declined slowly between the third (mean 88,082, SD 8790) and fourth (mean 80,556, SD 12,900) segments, and witnessed the lowest use in the fifth segment (mean 59,372, SD 6667). Compared to ambulatory care, VUC use peaked earlier in the first segment (mean 7100, SD 6920), had overall been in decline, but saw increases during the third (mean 6180, SD 1594) and fifth (mean 5092, SD 1712) segments compared to immediate previous segments. The IQR values showed that overall, variations in visits per month data shrunk in later time segments, which was particularly noticeable in the fourth time segment for urgent care (IQR 87) and the fifth segment for ambulatory care (IQR 7502). Levene test results on visits per month among time segments found the variance to be significant for in-person visits (*F*_4,21_=3.28, *P*=.03) and urgent care visits (*F*_4,21_=17.02, *P*<.001) but not for ambulatory care visits (*F*_4,21_=2.55, *P*=.07).

**Table 5:** Telemedicine use per month for non–COVID-19 suspected cases by time segment.

Visit type per month	First segment (until April 2020)				Second segment (May-September 2020)				Third segment (October 2020-February 2021)				Fourth segment (March-September 2021)				Fifth segment (October 2021-February 2022)		
	Mean (SD)	Median	IQR	Mean (SD)		Median	IQR	Mean (SD)		Median	IQR	Mean (SD)		Median	IQR	Mean (SD)		Median	IQR
In person	262,761 (15,630)	300,595	16,9756	269,518 (90,160)		299,436	33,424	311,069 (28,209)		310,285	5,289	369,679 (21,669)		376,610	41,208	350,946 (18,623)		353,797	28,118
Ambulatory care	58,457 (87,918)	23,360	80,374	113,834 (41,001)		99,126	48,358	88,082 (8790)		91,066	14,275	80,556 (12,900)		76,402	11,625	59,372 (6667)		57,845	7502
VUC^a^	7100 (6920)	6140	10,633	4596 (888)		4598	1206	6180 (1594)		5614	2428	4233 (613)		3988	87	5092 (1712)		4712	1744

^a^VUC: virtual urgent care.

### Trends in Telemedicine Service Use by Age Group

Table 6 decomposes telemedicine use by age group in our data. Across virtual visit types, the 25-34–year age group accounted for the largest proportion of telemedicine visits, peaking at 40,251 (16.74%) in the month of April 2020. This pattern of higher telemedicine use for those aged 25-34 years was even stronger for VUC visits, where this age group was responsible for a total of 112,247 (38.03%) urgent care visits in the entire period. The use of telemedicine for urgent care needs was the lowest for children and young adolescents aged less than 15 years (n=7420 visits, 2.56%) despite this age group being among the largest in the NYC population [21]. The distribution further showed that although telemedicine adoption for nonurgent care needs was relatively evenly distributed across age groups, patients between 25 and 44 years old were responsible for a disproportionate share of telemedicine-based urgent care visits.

**Figure 4:**
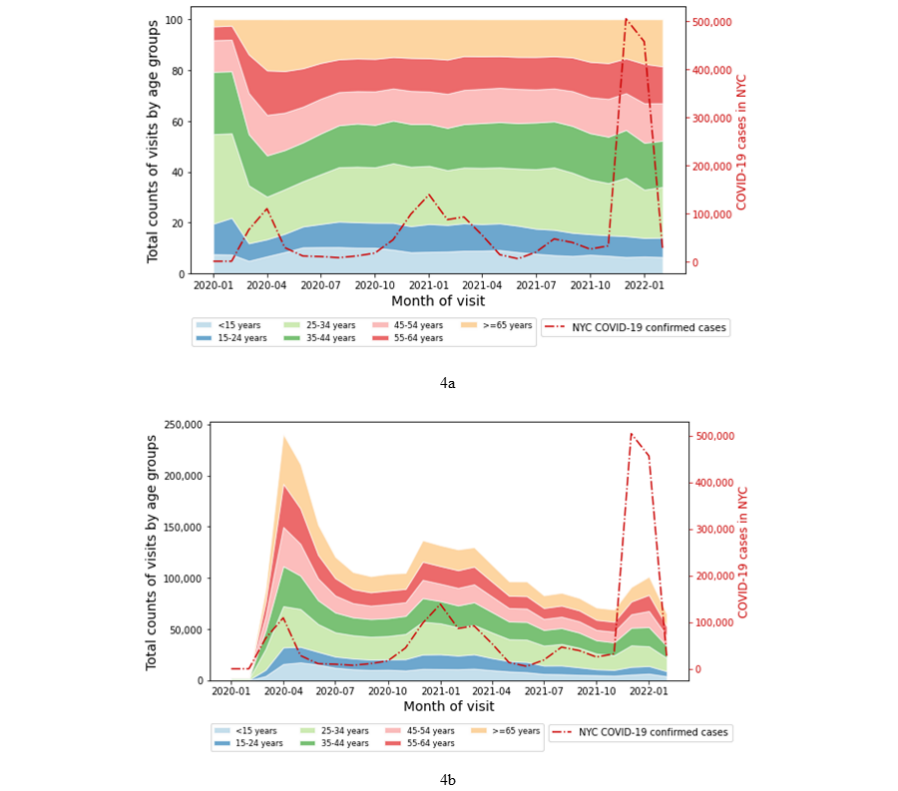
Trends in telemedicine use by age group. (a) Stacked area graph of percentage of visits and (b) total counts of visits. NYC: New York City.

Figures 4a and 4b illustrate the trends in virtual health visits among different age groups throughout the entire period considered in this study. Figure 4b shows that telemedicine adoption was the highest at the beginning of the pandemic (between March and May 2020) for most age groups. Although the number of telemedicine visits by the largest contributing age group (25-34 years) decreased from its peak of 40,251 per month in April 2020 to 12,948 in February 2022, the average visits per month (n=21,949, 21.34%) remained consistently higher than the prepandemic level of 1062 in February 2020. Figure 4a shows that although telemedicine use of the largest contributing age group (25-34 years) was high throughout the period after April 2020, the other age groups’ use of telemedicine grew each time the number of COVID-19 cases surged. Our analysis also found that patients from the 65 years and older age group remained consistent users of telemedicine (maximum 20,799, 15.21%, visits per month; minimum 11,927, 16.75%, visits per month) from June 2020 to February 2021. Overall, the distribution of telemedicine visits among age groups (Table 6) showed that although the use of telemedicine for nonurgent care among older patients increased (n=413,517, 15.37%) relative to prior reports [16], the use of telemedicine for urgent care remained quite low (n=11,630, 4.01%).

**Table 6:** Percentage distribution of telemedicine visits by age group and baseline population figures in NYC^a^ from the US Census Bureau data of 2020.

Age (years)	Population in NYC (%)	All telemedicine care (%)	Urgent care (%)	Nonurgent care (%)
<15	17.53	8.14	2.56	8.71
15-24	11.66	10.42	15.88	9.71
25-34	17.81	21.34	38.03	19.40
35-44	13.64	16.61	20.93	16.15
45-54	12.54	14.14	11.18	14.52
55-64	11.87	13.98	7.40	14.76
65 and above	14.95	15.37	4.01	16.75

^a^NYC: New York City.

### Patient Satisfaction

**Figure 5:**
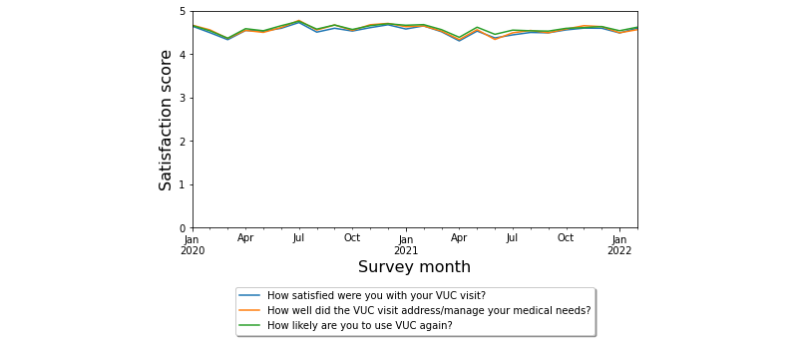
Trends in patients’ satisfaction with VUC visits (Q-Reviews) during the COVID-19 pandemic. VUC: virtual urgent care.

In total, 13,669 patients who used VUC across the 26-month study period responded to the satisfaction survey. Despite the inexperience of providers who adopted telemedicine rapidly, patients’ satisfaction with VUC remained unchanged during the acute pandemic phase (pre–COVID-19: n=847, 6.20%, mean satisfaction 4.38/5; acute COVID-19: n=1693, 12.39%, mean satisfaction 4.38/5). Q-Reviews data on patients’ satisfaction indicated that patients were consistently highly satisfied (n=13,669, 100%, mean satisfaction 4.53/5, minimum=4.31, maximum=4.78) with VUC visits (see Figure 5), despite 2173 (15.9%) patients reporting technical issues. In addition, 10,719 (78.41%) patients were highly satisfied with their VUC visits, and only 856 (6.26%) patients were least satisfied. More than 74% of patients (105 of 141) felt they saved at least an hour of time (including travel time) by using virtual care services and would likely recommend the services to a friend or colleague.

Finally, average video visits per patient increased from 0.013 in the prepandemic baseline to 0.827 between June and February 2020, before experiencing a slight decline, and then stabilized at 0.588 between June 2021 and February 2022. During the same periods, the average number of in-person visits slightly declined from 2.928 to 2.670 at first, followed by a steady increase to the prepandemic level of 2.894 per patient.

## Discussion

### Principal Findings

The COVID-19 pandemic prompted many health care systems to rapidly expand telemedicine services in response to significant disruptions in in-person care provisions [8,9]. However, the extent to which higher rates of telemedicine use have been maintained in the subsequent period is not yet clear. This study, which evaluated the extent to which telemedicine use has been sustained throughout the pandemic in 1 large health system in NYC, shows that although the early pandemic catalyzed rapid growth in telemedicine use, the inverse relationship between the volume of telemedicine visits and in-person emergency department visits [8] continued in subsequent periods of the pandemic, for both COVID-19–related care needs as well as routine care, such as preventive medicine, chronic condition management, and ambulatory specialty care. These findings suggest that the transition to telemedicine use in the manner of care delivery is at least partially lasting and not bounded by the end of the COVID-19 pandemic.

The detected change points in the time series data mostly coincided with the emergence of new variants and subsequent surges in COVID-19 cases in the United States. The first and second time segments match the timeline of the acute pandemic phase and the postacute pandemic phase from prior reports [8], while the subsequent 3 segments have overlaps between the timeline of the Delta and Omicron surges reported in the United States [22]. The analysis of time segments in general supports the characterization that telemedicine is part of the new norm in health care delivery. Specifically, telemedicine-based urgent care services increased alongside decreases in in-person emergency room visits with each recurring pandemic wave; these data substantiate the critical role of telemedicine in expanding emergency care capacity and services during a period of significant emergency service strain. In the ambulatory care setting, our evidence suggests a delayed, but even more pronounced, shift to telemedicine. Overall, although urgent care visits opened the door for wider adoption of telemedicine during the pandemic, it is nonurgent video visits that are currently driving the continued prevalence of telemedicine use. The analyses of visit trends from the time segments further suggest that although the use of telemedicine for both urgent care and ambulatory care services has gradually declined since its peak in the acute pandemic phase, the simultaneous decrease in variations in the monthly visit distribution further hints that telemedicine use is heading toward an equilibrium phase. Our result also suggests that during the latest Omicron wave, the use of telemedicine-based urgent care and ambulatory care demonstrated contradictory trends; although visits to urgent care for both COVID-19 and non–COVID-19 suspected cases increased, the use of ambulatory care decreased. Despite the massive increase in COVID-19 cases, why the use of ambulatory services decreased needs to be further investigated, and any potential barriers to wider adoption of virtual-ambulatory services need to be identified.

The trends in telemedicine visits suggest a proportionately larger role for urgent care facilities, particularly for COVID-19 care. Although the correlation between urgent care visits and COVID-19 confirmed cases was lower in the last wave of the pandemic (Omicron variant), we posit this result is due to the lower risk of severe outcomes from Omicron infection than in previous waves, especially the Delta variant [22,23]. With the emergence of new COVID variants [24], and the strong correlation between VUC visits and COVID-19 confirmed cases, the demand for VUC is not expected to decrease in the near future. More importantly, our observation of a new pattern of sustained demand for nonurgent, non–COVID-19–related telemedicine has enormous implications for health care delivery and equity. For patients, the high and steady level of satisfaction with virtual visits indicates their acceptance and willingness to persist with telemedicine services in the future. Whether broad reimbursement of virtual visits will continue [25] will be 1 of the factors determining the future of telemedicine as a mainstream mode of health care delivery in the United States. Analysis of patients’ demographics shows telemedicine use, especially for urgent care, was more frequent among younger patients. Combined with high satisfaction among telemedicine-based urgent care users, this indicates their acceptance and willingness to persist with telemedicine services in the future. Lower rates of telemedicine adoption among older adults may be due to their preference for emergency department visits, lower rates of technology adoption [26], and other reasons. Although recent reports suggest that smartphone adoption and internet use have more than doubled in the past 7 years among older adults [27], there remains a notable digital divide between younger and older Americans in telemedicine use. This divide is further skewed in the use of telemedicine for urgent care needs. Whether increased technology adoption will translate into telemedicine use uptake among seniors remains to be seen.

Furthermore, combined telemedicine and in-person visits increased by 18% from the pre–COVID-19 era (2019) to recent times (2021), and telemedicine was responsible (106%) for this increase, which suggests that virtual care delivery supplements rather than replaces in-person care. This may be a consequence of the enhanced access to care that telemedicine provides, allowing people with geographic, logistic, or other barriers to in-person care to more regularly access care. Telemedicine may be unlocking unmet needs of underserved patient populations and may potentially improve health equity and reduce health disparities if made accessible to inclusive populations. Although prior studies have found evidence that telemedicine access disparities mirror those in in-person health care access [16], whether telemedicine access disparities have reduced over time remains to be investigated. Nonetheless, evidence suggests health care organizations need to allocate additional resources to telemedicine, which should not come at the expense of in-person care. For providers, the transition means quickly developing and adjusting skills in virtual rapport building, empathy, diagnosis, and counseling.

### Comparison With Prior Work

To the best of our knowledge, this is 1 of the first studies to explore the longitudinal trends in telemedicine use throughout the pandemic. Other studies have explored various aspects of telemedicine during the COVID-19 pandemic, particularly its impressive expansion during the earliest phases. In 1 of the first case studies on telemedicine’s early growth, Mann et al [8] and Sherwin et al [28] described the exponential growth of telemedicine visits within our health system during the first wave of the pandemic, outlining the health system’s operational response as well. This work is complemented by a large volume of telemedicine-specific publications in 2020 and 2021, with the majority reporting on data and experiences from the early 2020 period (a review of PubMed literature on “telemedicine adoption” and “COVID-19” returns over 8000 papers, including case studies, opinion pieces, and reviews, from both US health systems and worldwide). Importantly, a number of articles on telemedicine during COVID-19 have called attention to new or growing disparities in the access and use of this technology, and its impact on health inequity [16,29-31]. A related systematic review on the use of digital health tools during the pandemic by Golinelli et al [31] revealed growth in the use of numerous digital health tools, including wearable devices, artificial intelligence (AI)–supported computing and clinical decision support, blockchain technology, and the internet of things (IOT), largely for the purposes of diagnosis, managing, and monitoring COVID-19–related disease. Our findings contribute to this growing body of literature by expanding our understanding of the longitudinal patterns of telemedicine use and its potential sustainable impact on care delivery.

### Limitations

Although there are many strengths of this study, we note the following limitations that can be addressed in future research. First, we used keyword matching to identify COVID-19 suspected cases from the diagnosis data, and the list of keywords were limited to the most common COVID-19–related symptoms to minimize the number of false-positive identifications. Additionally, most keywords were related to respiratory issues, which were the most common symptoms during the early waves of COVID-19 [32]. More recent studies have reported nonrespiratory symptoms of COVID-19 [33-36] that we incorporated, but we were unable to use a more accurate method, such as COVID-19 test results to evaluate how the recurring pandemic waves relate to telemedicine use. Additionally, satisfaction data were only available for VUC visits. Although we currently do not have similar systemwide patient satisfaction data for ambulatory care, recent reports from our maternal-fetal medicine practices suggest high satisfaction among patients who used telemedicine for nonurgent care, corroborating our findings [21]. Our data may not generalize to all contexts. For example, remote and rural patient populations were not well represented. Finally, with respect to demographics, we reported telemedicine usage by age group without correcting for the baseline proportion of the population in each age group, which may not be evenly distributed. In addition, this study did not consider any demographics other than age when evaluating patient populations that are telemedicine users. Prior studies have reported evidence of disparities for Black, male patients when accessing telemedicine [16]. Future research should consider race, gender, socioeconomic status, and geographic location when evaluating the demographics of telemedicine adopters.

### Conclusion

In conclusion, data show that the transition to telemedicine care in major health care systems prompted by the early phases of the pandemic has been sustained throughout the later phases of the pandemic [37]. This has been driven by a variety of telemedicine care seeking, including urgent care, primary care, and ambulatory specialty care, as well as both COVID-19–related and non–COVID-19–related complaints. Those most likely to use telemedicine are younger patients, with patients reporting high levels of satisfaction with telemedicine-based services. Overall, this suggests that telemedicine-based care has high acceptability for patients and potential sustainability as an important modality of care delivery. More research is needed to understand patterns of telemedicine use across different types of health systems, patients, and health concerns, as well as addressing ongoing challenges in telemedicine access and equity.

## Appendix

Multimedia Appendix 1 Overall trends in telemedicine and in-person visits in the 26-month period.

Multimedia Appendix 2 Data on telehealth and in-person visits for COVID-19 suspected and non-COVID-19 cases between January 2020 and February 2022.

## Abbreviations

EHR: electronic health record
ICD-10: International Classification of Diseases 10th Revision
NYC: New York City
NYU: New York University
NYULH: New York University Langone Health
VUC: virtual urgent care

## Data Availability

The primary data on visit records used for analysis came from the New York University (NYU) Langone Health (NYULH) system’s electronic health record (EHR) patient data containing protected health information (PHI) and cannot be made public. We included tabularized visit data per month in Multimedia Appendix 2. In addition, a partial de-identified version of the visit-level data might be available from Cosmos, Epic’s de-identified Health Insurance Portability and Accountability Act (HIPAA)–limited patient database. The data on COVID-19 numbers in New York City (NYC) are available as open source in Lau et al [9].
