# Supplementary figures and images for: Evidence for Telemedicine’s Ongoing Transformation of Health Care Delivery Since the Onset of COVID-19: Retrospective Observational Study
Source: JMIR Form Res. 2022 Oct 14;6(10):e38661. doi: 10.2196/38661 (PMC9578517; doi:10.2196/38661)

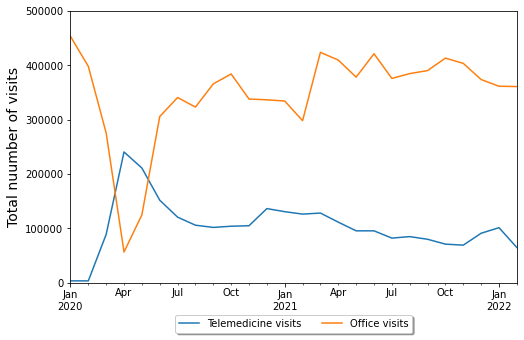

Supplement: Multimedia Appendix 1 [file formative_v6i10e38661_app1.png]
