# Supplementary material for: Evidence for Telemedicine’s Ongoing Transformation of Health Care Delivery Since the Onset of COVID-19: Retrospective Observational Study
Source: JMIR Form Res. 2022 Oct 14;6(10):e38661. doi: 10.2196/38661 (PMC9578517; doi:10.2196/38661)
Supplement: Multimedia Appendix 2 [file formative_v6i10e38661_app2.docx]

**Multimedia Appendix 2**

**Table S1.** Data on telehealth and in-person visits for COVID-19 suspected and non-COVID-19 cases between January 2020 and February 2022.

| **Month Year** | **In-Person Visit counts** | | **VUC visit counts** | | **Non-urgent telehealth visit counts** | |
| --- | --- | --- | --- | --- | --- | --- |
|  | COVID-suspected | Non-COVID | COVID-suspected | Non-COVID | COVID-suspected | Non-COVID |
| Jan’20 | 52637 | 400403 | 791 | 1213 | 492 | 719 |
| Feb’20 | 42574 | 355274 | 851 | 1334 | 287 | 722 |
| Mar’20 | 29298 | 245917 | 15817 | 14908 | 11990 | 45998 |
| Apr’20 | 6875 | 49450 | 9056 | 10947 | 33964 | 186389 |
| May’20 | 12131 | 112434 | 2785 | 5691 | 24781 | 177567 |
| Jun’20 | 23139 | 282313 | 1667 | 3486 | 15913 | 130579 |
| Jul’20 | 24822 | 315737 | 3485 | 4598 | 13234 | 99126 |
| Aug’20 | 23497 | 299436 | 6576 | 5207 | 11621 | 82221 |
| Sep’20 | 27965 | 337674 | 5561 | 4001 | 12311 | 79678 |
| Oct’20 | 30568 | 353290 | 5227 | 4340 | 12924 | 81266 |
| Nov’20 | 26186 | 311546 | 9454 | 5614 | 13079 | 76485 |
| Dec’20 | 26054 | 310285 | 14533 | 8012 | 18095 | 95541 |
| Jan’21 | 27777 | 306257 | 13109 | 7682 | 18645 | 91066 |
| Feb’21 | 23975 | 273969 | 8121 | 5254 | 16627 | 96056 |
| Mar’21 | 33697 | 389929 | 7422 | 5323 | 17238 | 97965 |
| Apr’21 | 32710 | 376610 | 3716 | 4047 | 15455 | 87996 |
| May’21 | 30642 | 347374 | 2544 | 3849 | 12475 | 76402 |
| Jun’21 | 32442 | 388582 | 2481 | 3960 | 12454 | 76371 |
| Jul’21 | 29760 | 345902 | 2814 | 3988 | 11056 | 64048 |
| Aug’21 | 30706 | 353797 | 4192 | 5009 | 11838 | 63599 |
| Sep’21 | 32003 | 358104 | 3875 | 4712 | 10422 | 60733 |
| Oct’21 | 35952 | 377043 | 3201 | 4191 | 7862 | 55535 |
| Nov’21 | 34773 | 368540 | 3383 | 3818 | 7924 | 53792 |
| Dec’21 | 33780 | 339788 | 11463 | 8192 | 13330 | 57845 |
| Jan’22 | 32518 | 328732 | 6715 | 6489 | 16195 | 71654 |
| Feb’22 | 30067 | 330619 | 1896 | 3236 | 6992 | 52446 |
